# Coronavirus Disease and Cardiovascular Disease: A Literature Review

**Published:** 2021-03-24

**Authors:** Vijaya Krishna Prasad Vudathaneni, Swetha Bharathi Nadella, Rama Brahmam Lanke, Ramanarayana Boyapati

**Affiliations:** ^1^Physician, North Central Bronx Hospital, 3424 Kossuth Ave, Bronx; ^2^Resident Physician, James J Peter VA Medical center, 130W Kingsbridge Road, Bronx 10468, New York; ^3^Associate General Dentist, C/O Familia Dental LLC, 3200 Andrews Hwy, Ste 400, Midland Texas, United States; ^4^Department of Periodontology, Sibar Institute of Dental Sciences, Guntur, Andhra Pradesh, India

**Keywords:** myocardial injury, severe acute respiratory syndrome coronavirus 2, thromboembolism

## Abstract

**Background and Aim:**

Although severe acute respiratory syndrome coronavirus 2 primarily affects the respiratory system, involvement of cardiovascular system is not uncommon and a range of cardiac manifestations among Coronavirus Disease (COVID-19) patients were reported in the literature. Furthermore, it is evident from scientific literature that the incidence of deaths and hospitalizations has been increasingly more among COVID-19 subjects with pre-existing cardiovascular disease (CVD). Various pathophysiological mechanisms have been proposed to explain the cardiovascular involvement in COVID-19. Another emerging significant concern is the varying presentations of COVID-19 and side effects due to the medication used in the management of COVID-19 patients. This review attempts to provide a comprehensive overview of the existing literature on the possible association between CVD and COVID-19 with emphasis on the pathophysiological mechanisms, cardiac manifestations, and impact of medications used for COVID-19 on cardiovascular health. Based on the available literature, we conclude that though CVD could not be reckoned as an independent risk factor for COVID-19 infection, it is evident that pre-existing CVD has an influence on the severity of COVID-19 infection and associated mortality.

**Relevance for Patients:**

Literature suggests that people with pre-existing CVD are at increased risk for COVID-19 and associated severity. Consequently, it becomes important to thoroughly gain insights into the possible pathophysiological mechanisms, cardiac manifestations in COVID-19, and the impact of COVID-19 treatment on the cardiovascular system.

## 1. Introduction

According to the World Health Organization reports, 79.2 million confirmed Coronavirus Disease (COVID-19) cases were detected and nearly 1.75 million deaths were registered across the world as on December 27, 2020 [1]. As there has been a consistent daily increase in the number of COVID-19 confirmed cases in many countries, these numbers may worsen soon and the disease may continue to impose significant burden on the health-care delivery systems worldwide. Severe acute respiratory syndrome coronavirus 2 (SARS-CoV-2), the virus causing COVID-19, primarily affects the respiratory system though the involvement of other organs and systems is not uncommon. The clinical presentations of the disease are too heterogeneous ranging from mild fatigue to severe hypoxia with respiratory failure [2]. This resulted in the categorization of the severity of COVID-19 by Wu and McGoogan [3] and the Society of Pediatrics, Chinese Medical Association [4].

SARS-CoV-2 is an enveloped, single-stranded RNA virus belonging to Coronaviridae. HKU, 229E5, and hCoV-0C43 are among the mild common cold causing coronaviruses affecting human beings. However, SARS-CoV and Middle East respiratory syndrome coronavirus MERS-CoV are the recently emerged pathogenic coronaviruses, infecting humans, with 8000 and 2500 reported cases worldwide, respectively [5,6]. The major route of transmission of SARS-CoV-2 is through respiratory droplets. The secondary attack rate for COVID-19 ranged from 0.5 to 5% [7,8]. Incubation periods for the disease did not differ significantly between people with the 95% confidence interval of the median incubation period for SARS-CoV-2 being 4.5–5.8 days and a vast majority of the affected demonstrating symptoms within 12 days from the time of exposure [9]. Shi *et al*. reported that <20% of COVID-19 affected subjects had significant symptoms such as dyspnea, tachypnea, and hypoxemia. Symptoms of critical COVID-19 infections include respiratory failure, multi-organ failure, and sepsis induced hypotension [10]. Development of cardiovascular disorders was reported in some COVID-19 cases and the literature suggests that people with pre-existing cardiovascular disease (CVD) are at increased risk for COVID-19 and associated severity [11]. With this background, the objective of this review is to provide a comprehensive overview of the association between COVID-19 and CVD.

## 2. CVD and Coronavirus: an epidemiological perspective

CVD has been identified as a common comorbidity among patients infected with viruses belonging to the Coronavirus family. The prevalence of CVD among SARS patients was 8% and is reported to be a significant contributor for the increased risk of death among those affected with SARS [12,13]. With regard to MERS, hypertension and CVD were prevalent among nearly 50% and 30% of the affected subjects, respectively [14]. This association is apparently evident in the context of COVID-19 as well with presence of cardiovascular comorbidities among the COVID-19 affected at an increased frequency. It was hypertension as a comorbidity and 17% of COVID-19 confirmed cases had coronary heart disease [15]. Yang *et al*. conducted a meta-analysis to document the prevalence of comorbidities among COVID-19 patients and found that hypertension, diabetes mellitus, and CVD are among the common comorbidities among the infected patients. It was reported that the 95% confidence interval for the prevalence of CVD was 4–7% [16]. The presence of underlying cardiovascular comorbidities was also found to be associated with increased mortality among COVID-19 infected. In a study conducted by Wang *et al*. in Wuhan, China, 31% of the COVID-19 cases had hypertension and this percentage increased to 58% when patients admitted in ICU were exclusively considered [17]. Similarly, the prevalence of hypertension and coronary heart disease was 30% and 8% in another study conducted by Zhou *et al*. in China [18]. Although the exact mechanism leading to these observations is not known, the interference of age as a confounding factor in the association between CVD and COVID-19 cannot be ignored, with older people demonstrating high prevalence of CVD and increased susceptibility of a symptomatic COVID-19 infection due to a relatively weaker immune system among the elderly.

## 3. Pathophysiological mechanisms

The elevated expression of several cardiovascular biomarkers has been reported in severe COVID-19cases [17-19]. These changes support involvement of the cardiovascular system, which can be explained by the combined effects of several mechanisms.

### 3.1. Renin-angiotensin system (RAS) imbalance

Angiotensinogen is converted to angiotensin I (Ang-I) by the protease rennin, which consequently gets converted by Angiotensin Converting Enzyme (ACE) to angiotensin II (Ang-II). ACE2 facilitates the conversion of Ang-II to angiotensin-(1–7), by removal of carboxy-terminal phenylalanine, which on binding with the MAS receptor results in vasodilation and anti-inflammation. However, it is scientifically established that to facilitate the fusion with host cells, SARS-CoV binds to human ACE 2 with the amino terminal of its spike protein [4]. SARS-CoV2 being a coronavirus that belongs to the b genus may also have ACE2 as its binding receptor. The expression of ACE2 on cells is downregulated in SARS-CoV infection leading to disruption in the physiological balance between ACE/ACE2 and Ang-II/angiotensin-(1–7). This marked downregulation of ACE2 and the corresponding upregulation of Ang-II may aggravate perpetuate cardiac injuries [20]. High Ang II levels in plasma among COVID-19 patients adds strength to the supposition that SARS-CoV2 binds to ACE2 resulting in the elevated production of Ang II through RAS. This increases burden on the cardiovascular system as the heart load increases leading to cardiomyocyte hypertrophy and hypertension [21].

### 3.2. Overactivation of the immune system

Replication of virus following the host cell fusion and this invasion of alveolar surfaces would result in alveolitis and the invasion of the cardiac muscle cells results in edema and lysis of the cardiomyocytes, which further lead to release of pro-inflammatory cytokines [22]. The deposition of pro-inflammatory cytokines and the filtration of inflammatory cells at the site of injury causes “inflammatory storms” which warrants an overactivation of the immune system. This leads to damage of cardiomyocytes and decreases the adherence of the atherosclerotic plaques in coronary vessels. All these mechanisms predispose to cardiovascular events [22]. Huang *et al*. reported the cytokine profiles of COVID-19 infected subjects with elevated Interleukin (IL)-2, IL-7, interferon- g, and tumor necrosis factor a [11]. Virus associated was reported to be associated with increased COVID-19 fatality in a multicentric study among infected subjects in Wuhan, China, where significant differences in ferritin levels and IL-6 were between survivors and non-survivors [19]. In autopsy findings, cytokine storm induced by the inflammatory response syndrome to COVID-19 was discussed to have resulted in cardiac interstitial mononuclear inflammatory infiltrates [23,24].

### 3.3. Decreased partial pressure of oxygen in blood

Damage to alveolar cells in COVID-19 patients results in hypoxemia. As the partial pressure and saturation of oxygen falls consistently there is deposition of oxygen free radicals, which on circulation across the body damages the cardiomyocytes. Another mechanism is the activation of NADPH oxidase enzymatic complex by the downregulation of ACE2 and corresponding upregulation of Ang-II in COVID-19. Ang-II binds to Angiotensin Type I Receptor (AT1R) which stimulates the activity of NADPH oxidase that reduces oxygen to superoxide. While clear underlying mechanisms for stimulation of NADPH oxidase are complex, it is known to occur at the genetic, transcriptional, and post-transcriptional levels involving multitude of signaling molecules and scaffolding proteins [25]. ACE2 deficiency was shown to increase NADPH oxidase activity and resulted in oxidative stress in the study conducted by Wysocki *et al*. [26]. To accommodate for the metabolic demands, blood flow intensifies increasing the individual’s risk for heart failure. Furthermore, hypoxemia is an independent predecessor for inflammatory response that promotes “inflammatory storm” and contribute toward incidence of cardiovascular events [27].

### 3.4. Elevated catecholamine levels in plasma

An initial response to viral invasion is the activation of immune system, and the interaction between viral and host cells is a complex phenomenon. Besides, stimulation of a1-adrenoceptors by catecholamines can reinforce the vasoconstriction caused by excess Ang-II as a result of downregulation of ACE2 and could contribute toward a significant increase in vascular resistance. Moreover, catecholamines stimulate b1-adrenoceptors in the kidney to increase renin secretion that consequently increases Ang-II resulting in further deterioration of cardiovascular health. Response from the emotional perspective such as anxiety, fear also lead to increased production of catecholamines the concentration in plasma of which is directly related to hypertension, coronary artery disease, and cardiac failure. Increased level of catecholamine levels in plasma also has the implication of myocardial toxicity that could result in circulatory disturbances and arrhythmias [26]. Although a direct study of catecholamines among COVID-19 subjects could offer useful insights, the scope for these observations is hampered by severe illness such as respiratory failure, compromised cardiovascular health, and shock and more so among those on adrenaline infusion.

## 4. Cardiovascular manifestations in COVID-19

Although it is known that SARS-CoV 2 primarily affects the respiratory system, the negative impact COVID-19 has on cardiovascular health cannot be overstated. It has been thoroughly established in literature that cardiac manifestations contribute significantly towards COVID-19 case fatality rates [27-29]. The following are the most common cardiac manifestations observed among COVID-19 patients.

### 4.1. Myocardial injury

Regardless of the presence or absence of respiratory symptoms, it has been observed that ischemic and non-ischemic myocardial injury is one of the significant outcomes of COVID-19 infection [30,31]. The incidence of myocardial injury as a COVID-19 manifestation varied between 7% and 28% among hospitalized COVID-19 patients in the scientific literature [32]. High spikes in troponin levels were identified in patients with severe disease. In a cohort study conducted by Shi *et al.*, nearly 20% of the hospitalized patients demonstrated cardiac injury. This finding gains significance in light of the fact that COVID-19 fatality rate is 10 times higher among patients with cardiac injury compared to those who do not have myocardial injury [33]. Zhou *et al*. reported in their multicentric study that the proportion of myocardial injury among COVID-19 non-survivors was 46% as compared to 1% among survivors [18].

### 4.2. Heart failure

Among COVID-19 patients with previous history of cardiovascular problems and undiagnosed heart diseases, heart failure may occur [34]. It is imperative to focus on the findings reported by Zhou *et al.*, where the proportion of heart failure was 52% among non-survivors as compared to 12% among survivors [18]. These findings corroborated with those reported by Chen *et al.*, where heart failure was notice among 49% and 3% of the non-survivors and survivors, respectively [35]. It is also reported in the literature that COVID-19 patients with the previous heart failure could possibly experience an acute decompensation [36].

### 4.3. Cardiac arrhythmias

Arrhythmias are another common manifestation among COVID-19 patients. While progressive arrhythmias among infected individuals could be reckoned as an indication for cardiac involvement, it is not uncommon for arrhythmias to be the initial presentation of COVID-19. It was postulated by Guo *et al*. that high TnT levels have an increased incidence of malignant arrhythmias [37]. Palpitations were reported to be among the most common clinical presentations of COVID-19 in a study conducted by Liu *et al*. in Wuhan, China [38]. Although the reasons for palpitations and the nature of arrhythmias were not mentioned by Wang *et al.*, they reported increased frequency of arrhythmias among COVID-19 patients in ICUs compared to those who did not require intensive care [17].

### 4.4. Thromboembolism

It has been thoroughly established in the literature that COVID-19 activates the coagulation cascade. In a study conducted by Han *et al.*, a significant reduction in circulating antithrombin III, substantial increases in the fibrinogen and D-dimer levels were reported among COVID-19 patients as compared to healthy controls. Severe inflammation in COVID-19 is also marked by higher platelet counts compared to non-COVID-19 patients with pneumonia [39]. Tang *et al*. reported higher incidence of disseminated intravascular coagulation (DIC) among the deceased compared to the survivors, and a short duration of 1–12 days was identified between the time of hospitalization and the onset of DIC [40]. Literature suggests that almost a quarter of COVID-19 patients had deep venous thrombosis [41]. The rationale for this observation could be elevated levels of D-dimer, fibrinogen among COVID-19 affected [39]. An association between severity of COVID-19 and the levels of these coagulation markers was also reported by Xiong *et al*. in a meta-analysis [42]. Any level of D-dimer more than 1mg/ml was reported to be a contributing factor for in-hospital deaths by Zhou *et al*. [18]. The incidence of venous thromboembolism as a COVID-19 manifestation varied between 20% and 43% among COVID-19 patients requiring intensive care in spite of preventive anticoagulation. To prevent incident thrombophlebitis among COVID-19 patients, heparin is recommended by the WHO, due to its pleiotropic properties, for hospitalized COVID-19 patients [43]. Yin *et al*. reported improved prognosis with heparin use among hospitalized COVID-19 patients with elevated D-dimer levels [44].

## 5. Cardiac consequences of COVID-19 treatment

Varying presentations of COVID-19 and side effects due to the medication used in the management of COVID-19 patients emerged as a significant challenge for physicians providing services for these patients. Among various pharmacological management strategies of COVID-19, the use of antiviral drugs, anti-malarial drugs, immunomodulatory medicines, glucocorticoids, and plasma from COVID-19 recovered patients is the common [45-47]. Chloroquine and hydroxychloroquine result in alkalinization of the intracellular phagolysosome. This leads to under-glycosylation of ACE2 receptors thus limiting the fusion of SARS-CoV 2 and the host cells [45]. Regardless of the concomitant use of COVID-19, hydroxychloroquine was found to be decreasing the viral carriage among the in-hospital COVID-19 affected [48]. However, these drugs prolong the QT interval and may result in fatal arrhythmias, especially in cases with electrolyte abnormalities [49]. Furthermore, thorough monitoring for hypotension and bradycardia is warranted among COVID-19 patients using chloroquine as the drug holds the potential for inhibiting CYP2D6 resulting in elevated levels of beta blockers [50]. The antiviral drugs used in the management of COVID-19 patients may result in bradycardia, prolongation of QT and PR interval [49,51,52] and may have an influence on the warfarin dosing [53]. This possibility of drug-drug interaction between antiviral medication and the anticoagulants is an area of concern. The side effects of remdesivir, which is established to be contributory toward faster recovery from COVID-19 infection [54], are increase in liver enzymes and respiratory failure [55]. Monitoring ECG parameters is extremely important in the context of COVID-19 pandemic in light of the fact that the medications used in the management of this disease and also the possible hypokalemia subsequent to diarrhea, which is one of the clinical features in COVID-19 affected, may prolong the QT interval increasing the risk for torsade Despointes.

Although there is uncertainty whether RAAS inhibition have any effect on the ACE2 expression [56,57], use of RAAS inhibitors emerged as an area of concern as ACE2 protein is used by SARS-CoV2 for cellular entry [58]. However, the general recommendation amidst uncertainty is to continue the use of RAAS inhibitors among stable COVID-19 patients [59]. The negative impact of cytokine release syndrome, among COVID-19 affected, on the cardiopulmonary system prompted the use of corticosteroids to curtail the overactivation of the immune system [60]. Nevertheless, use of corticosteroids is routinely avoided to decrease the risk of exacerbation of the prevailing lung damage [61]. Therefore, it has been recommended that screening of COVID-19 patients for ESR, platelet count, serum ferritin levels and obtaining a hemophagocytic response (H Score) be done in the identification of COVID-19 patients where immunosuppression could be necessary.

## 6. Conclusion

The notion of increased risk of COVID-19 infection among subjects with pre-existing CVD remains equivocal as the numbers suggest a comparable prevalence of CVD among COVID-19 infected and the general source population. Nevertheless, CVD apparently is associated with increased hospitalizations and death among COVID-19 cases. It is evident that pre-existing CVD may aggravate the COVID-19 disease course with the presentation of aforementioned cardiac manifestations. Moreover, the pathophysiological mechanisms involved in COVID-19 may lead to cardiomyocyte hypertrophy and damage, circulatory disturbances, and arrhythmias. Therefore, close monitoring of COVID-19 subjects for cardiovascular health is warranted and it is advisable for subjects with pre-existing CVD to be more careful in practicing the precautionary measures such as maintaining social distancing, avoiding unnecessary travel, following respiratory hygiene/cough etiquette to avoid COVID-19 infection.
